# Perceived Stress at Work and Associated Factors among E-Waste Workers in French-Speaking West Africa

**DOI:** 10.3390/ijerph19020851

**Published:** 2022-01-13

**Authors:** Nonvignon Marius Kêdoté, Ghislain Emmanuel Sopoh, Steve Biko Tobada, Aymeric Joaquin Darboux, Pérince Fonton, Marthe Sandrine Sanon Lompo, Julius Fobil

**Affiliations:** 1 Department of Health Environment, Regional Institute of Public Health Comlan Alfred Quenum, University of Abomey-Calavi, Ouidah P.O. Box 384, Benin; ghislainsop@gmail.com; 2 Community of Practice Ecohealth for West and Central Africa, University of Abomey-Calavi, Cotonou 03 P.O. Box 3975, Benin; tobada93@gmail.com (S.B.T.); darboux14@hotmail.com (A.J.D.); perincefont@gmail.com (P.F.); 3 Unité de Formation et de Recherche en Sciences de la Santé, Université Joseph KI ZERBO, Ouagadougou 03 BP 7021, Burkina Faso; sandrine2_sanon@yahoo.fr; 4 Department of Biological, Environmental and Occupational Health Sciences, School of Public Health, West Africa GEOHealth Network, University of Ghana, Legon, Accra P.O. Box LG 25, Ghana; jfobil@gmail.com

**Keywords:** perceived stress, work, associated factors, e-waste, West Africa

## Abstract

Perceived stress at work is an important risk factor that affects the mental and physical health of workers. This study aims to determine the prevalence and factors associated with perceived stress in the informal electronic and electrical equipment waste processing sector in French-speaking West Africa. From 14 to 21 November 2019, a cross-sectional survey was carried out among e-waste workers in five countries in the French-speaking West African region, and participants were selected by stratified random sampling. Participants were interviewed on socio-demographic variables and characteristics related to e-waste management activities using a questionnaire incorporating Cohen’s Perceived Stress Scale (10-item version). Factors associated with perceived stress were determined by multivariate logistic regression. A total of 740 e-waste workers were interviewed. The mean age of the workers was 34.59 ± 11.65 years, with extremes of 14 and 74 years. Most of the interviewees were repairers (43.11%). The prevalence of perceived stress among the e-waste workers was 76.76%. Insufficient income, number of working days per week, perceived violence at work, and the interference of work with family responsibilities or leisure were the risk factors that were the most associated with perceived stress. The high prevalence of perceived stress and its associated factors call for consideration and improvement of the working conditions of e-waste workers.

## 1. Introduction

The World Health Organization (WHO) defines perceived work stress as the set of reactions that workers have when faced with work constraints and work pressures that do not correspond to their knowledge and/or abilities and that challenge their ability to carry out the work assigned to them [1]. In the workplace, stress is an important risk factor for human health. It favors the occurrence of physical and mental health problems and negatively affects the performance of workers in carrying out their activities [1]. Understanding the risk factors associated with worker productivity loss has become an important area of study within the area of occupational health research [2]. Occupational stress is a result of the mismatch between an individual and the environment, as harmful physical and emotional responses occur when the requirements of the job do not match the capabilities, resources, or needs of the worker. Generally, a higher discrepancy between external stresses and an individual’s capabilities causes a higher level of stress [3].

The workload (quantity of work to be accomplished, including the intellectual requirements and the time constraints to be respected in the realization of the work) is known to be a major stressor in contemporary and fast-growing informal sectors such as e-waste management. Indeed, the e-waste sector in Africa is characterized by the poor enforcement of environmental standards and virtually no regulation [4,5]. In addition to this, the problem of e-waste management in developing countries lies in the lack of appropriate recycling methods and technologies to effectively extract the required components from used electronic devices while also minimizing the health risks to the workers who are involved in recycling this type of waste [6]. In western Africa, the quantity of e-waste that is imported is increasing rapidly. Between 2014 and 2019, outside of imports, the amount of e-waste generated increased from 1.9 to 2.9 million tons, and a significant portion of that waste was processed by workers in the informal sector [7,8].

In West Africa, especially in French-Speaking countries, evidence regarding the risk of stress among e-waste workers is limited. One study conducted in Ghana showed variable levels of stress and highlighted associations between exposure to adverse physical conditions and elevated heart rate among e-waste workers [9]. In the formal sector, it has been observed that occupational strain resulting from exposure to stressors in the workplace can affect the mental and physical health of employees [10]. Therefore, a study was conducted in five French-speaking West African countries to determine the prevalence and factors associated with perceived stress in the informal e-waste processing environment. The findings from this study may be important for the authorities of these countries and other key stakeholders who play a critical role in improving the working environment of informal e-waste workers.

## 2. Materials and Methods

The present study used data collected from a research project entitled “Assessment of health risk associated with the handling of waste electronic and electrical equipment (WEEE) in West Africa: a multi-country study”. In the section below, the data collection methods and the procedure used to obtain the sample of participants for this study are described.

### 2.1. Study Area

The data were collected in five French-speaking West African countries: Benin, Burkina Faso, the Ivory Coast, Mali, and Senegal. E-waste workers were recruited at their work sites, which were located in the economic towns in each of the countries being analyzed (Cotonou, Ouagadougou, Abidjan, Bamako, and Dakar).

### 2.2. Study Population

The study population consisted of e-waste workers operating in the economic towns of the selected countries. The targets of the study belong to three categories (recyclers, repairers, and collectors) of WEEE. The collectors are e-waste workers who are responsible for main tasks such as collection, sorting, and trading. For the repairers, these tasks are the dismantling, the removal of electronic wire coating for soldering, and troubleshooting. The recyclers’ tasks include dismantling, the removal of coating from electronic wires, the burning of electronic wires or WEEE, and the melting of lead–acid batteries.

E-waste workers who were conducting their activities in the informal sector and in recycling/repair workshops or in WEEE landfill located in the selected towns were also included.

### 2.3. Sampling

With a sampling framework obtained from an exploratory survey carried out before data collection in each country, sampling carried out based on stratum with probability proportional to the weight of each category of e-waste workers. A simple random draw was carried out within each activity category. If the targets refused, a new simple random draw was carried out to replace them with others from the same category during the same sampling frame.

The Schwartz formula was used to determine the number of participants that needed to be included in our survey. Respiratory problems were the most reported and studied conditions among e-waste workers. Therefore, the sample size by country was computed using the prevalence of respiratory problems occurring among e-waste workers in Chile (24.7%) [11]. The desired precision was set at 7%. The sample size was 161 per country, with an addition of 10% to cover non-response. Data were collected in each country and were aggregated to obtain the information of 800 e-waste workers in Benin, the Ivory Coast, Burkina Faso, Mali, and Senegal.

For the present study, a few respondents were excluded from the analysis because of non-response or due to abstention from answering certain items of the perceived stress scale questionnaire or other questions relevant to the present study. The sample size that was considered in the data analysis included 740 interviewees.

### 2.4. Data Collection

Data collection was conducted through a cross-sectional survey that was administered from 14 to 21 November 2019, with a questionnaire being administered through semi-structured interviews with e-waste workers. This questionnaire was validated in a previous study in the Agbogbloshie landfill site in Accra, Ghana, as part of the West Africa-Michigan Charter II for GEOHealth project [6]. Originally in English, it was translated into French and adapted for the project. It was digitized using the KoboToolBox application and was administered to the e-waste workers in French as well as in local languages for those who did not speak French. Data were recorded directly on tablets. Stress and its levels were assessed according to the Cohen’s Perceived Stress Scale questionnaire (10-item version) [12]. This scale assesses the frequency at which life situations are threatening, i.e., unpredictable, uncontrollable, and distressing. The items are rated from 0 to 3. The positive items, i.e., those numbers 4, 5, 7, and 8, are coded inversely to the other so-called negative items. The sum of the scores of the items makes it possible to determine whether stress is present or not as well as its level. The stress level is judged as “Null” (no stress) when the total score obtained is between 0 and 9, “Moderate” when the total score obtained is between 10 and 19, and “High” when the total score obtained is between 20 and 30.

### 2.5. Study Variables

For the present study, after a literature review, relevant variables were selected from the overall database. The selected variables were the socio-demographic characteristics of the participants (gender, age, marital status, country of origin, educational status, and daily income); the characteristics of the participants’ e-waste work (activity category, the number of working days per week, number of working hours per day), and risk factors for perceived work stress (burnout after work, exposure to hazardous physical conditions, violence at work, work interference with family responsibilities/leisure activities, supervisor support). The sum of the scores obtained for the items from Cohen’s Perceived Stress Scale questionnaire was used to assess the presence or absence of stress as well as the levels of perceived stress exposure.

### 2.6. Data Analysis

The data were analyzed using R Studio version 1.2.5001 (RStudio, PBC, Boston, MA, USA). In bivariate analysis, the stress variables were tested for dependence on other study variables using the Chi-2 test. In the multivariate analysis, the calculation of the Odds Ratio (OR) and its 95% confidence interval (95% CI) allowed the identification of risk factors related to perceived stress among e-waste workers. A backward stepwise multivariate logistic model was used to search for the risk factors. To construct the initial model, all of the variables with a *p*-value of less than 0.20 in the bivariate analysis with perceived stress were introduced. A significance level of 5% was defined and used.

## 3. Results

A total of 740 (97.16% males and 02.84% females) respondents were interviewed. The mean age of the participants was 34.59 ± 11.65 years old, with extremes of 14 and 74 years old. The most represented age group was that of 25 to 45 years old, which represented 57.30% of the participants. Collectors represented 34.73% of the respondents, recyclers represented 22.16%, and repairers represented 43.11%. Regarding their educational status, about 4.73% attended university, and 32.16% attended primary school. Most of them were married (38.51%). Table 1 presents more details about the sociodemographic characteristics of the participants.

In the bivariate analysis, inadequate income (*p* = 0.002), number of days worked per week (*p* = 0.001), workplace violence (*p* = 0.012), interference of work with family responsibilities, and/or leisure (*p* < 0.001) were significantly associated with perceived work stress. Age (*p* = 0.137), support from supervisor or co-workers (*p* = 0.110), burnout after work (*p* = 0.160), exposure to adverse physical conditions (*p* = 0.107), number of working hours per day (*p* = 0.115), although not associated with the 5% statistical threshold, were included in the initial multivariate model.

In the final multivariate model, insufficient income, number of working days per week, and perceived violence at work and interference of work with family responsibilities and/or leisure were the factors that were the most associated with perceived stress. The results of the multivariate analysis show that participants with insufficient income were 1.46 times more likely to be stressed at work than those with sufficient income (OR: 1.46; CI95: [1.03–2.08]; *p* = 0.036). Those who experienced violence at work were 3.07 times more likely to be stressed at work than those who did not experience violence (OR: 3.07; CI95: [1.21–10.40]; *p* = 0.036). Work interference with family/leisure responsibilities among e-waste workers resulted in a risk of feeling stressed at work that was 2.41 times greater than those whose work did not interfere with family or leisure responsibilities (OR: 2.41; CI95: [1.42–4.31]; *p* = 0.002). Those working seven days a week had a risk of stress that was 1.67 times greater than those who did not (OR: 1.67; CI95: [1.12–2.52]; *p* = 0.014). Table 3 presents more details from the multivariate analysis.

## 4. Discussion

The objective of this study was to determine the prevalence of perceived stress at work and the factors likely to explain its occurrence or presence among e-waste workers in French-speaking West Africa. The results obtained show a high prevalence of perceived stress (76.76%). In Ghana, Kploanyi et al. (2020) reported a prevalence of occupational stress of 32.8% among the employees of telecommunications companies [10]. The high prevalence of workplace stress observed in the present study could be explained by the bad working environment conditions in the informal e-waste sector. Several research works report that e-waste workers operate in a high-stress environment [13,14].

Regarding income, inadequate income was associated with higher levels of perceived stress among e-waste workers. Insufficient income degrades self-esteem and exposes workers to psychological disorders such as anxiety and depression. A worker with a low income is at a greater risk of developing work-related stress than a worker with a higher income [15]. A previous study reported that perceived stress was significantly higher among respondents with a low monthly disposable income [16]. This relationship between the presence of stress and insufficient income is explained by the multiplication of potential stressors since those with insufficient income may find themselves unable to meet their personal needs and those of their families in terms of nutrition, housing, and health. Furthermore, the association of low income with perceived stress may be explained by the observation that living with less income may be detrimental to one’s physical and mental health, as financial hardships create a context of stress [17]. According to Cheemah et al. (2021), high stress was significantly associated with low life satisfaction [18]. Similarly, Nguyen et al. (2021) found that waste collectors who reported insufficient income had a risk of mental distress that was 1.98 times higher than waste collectors who were in a better financial situation [19]. Being at the bottom of society’s hierarchy may lead to stress resulting from feelings of bitterness based on invidious social comparisons, and the perception of social inequality can be an incentive for health-risk behaviour [18].

The number of working days per week was associated with perceived stress. Recyclers and repairers generally work all seven days of the week. Repairers work six days per week [20]. The continuous work, the workload, and its arduousness accentuate exhaustion after work. According to Owona et al. (2019), post-work exhaustion and/or fatigue influence the occurrence of stress [15]. A previous report suggested the mechanism by which long working hours affect work-related stress. Long working hours reduce the time for sleep, recovery from work, and recreational activities. In addition, they increase job demands and time for exposure to workplace hazards. Therefore, workers with long working hours are more likely to report work-related stress [21]. Working every day of the week would increase burnout and, in turn, probably reduce the amount of sleep that one receives per night, which is a factor that is associated with perceived stress, as reported in research works [22]. Some authors have reported that workers with weekly working hours of 60 h or more are more likely to report high levels of stress [21].

Among the e-waste workers, workplace violence was associated with perceived stress. In a study conducted in Accra in 2016, more than a quarter (26%) of workers at a WEEE recycling site reported being subjected to violence during their activities [23]. Previous studies have reported workplace violence as an aggravating factor in perceived work stress [24,25]. This could be explained by the fact that workplace violence degrades professional and inter-human relations and creates a climate of insecurity. Moreover, job contexts in which violence appears increase employee stress, and this tension, in turn, increases the probability of such violence reoccurring [26]. According to Saleh et al. (2020), workplace violence is one of the workplace factors that can affect many aspects of an employee’s life outside of work [3].

Work interference with family responsibilities or leisure was also associated with perceived stress. A similar association was found among caregivers in Yaoundé [25]. Over-involvement and exceeding regular working hours and workload take a toll on family or leisure time. In Ghana, e-waste workers preferred agricultural or professional occupations that would allow them to stay close to their families [14]. Occupational stress is a permanent condition that is caused by workplace conditions that have an antagonistic influence on workers’ employment progress and overall prosperity [27]. Several empirical studies have reported that work–family conflict is associated with increased levels of stress [28]. A study on worktime reduction showed that shorter workdays have a positive impact on work–family interaction [29]. Work–family conflict is a special form of inter-role conflict that arises when there are incompatible demands between work and family roles. Therefore, it appears that work–family conflict has an indirect relationship with mental health through stress [30].

The present study proves that the working environment experienced by informal e-waste sector workers lacks protection, which poses the risk of stress to workers, thereby undermining their health and well-being. The inadequate working environment of the informal sector workers makes the social, spatial, environmental, and facilitating roles of planners in the informal sector crucial. With the integration of informal sector issues into public policy, including in public health policy, and the contribution of research, the informal sector could be sufficiently guided to appreciate the opportunities and challenges, allowing sustainable solutions to the inherent health and safety problems posed by these environments to be found. Therefore, there is a need for governments to formulate a national occupational health and safety policy and regulatory framework to regulate the informal sector [31]. As a limitation, this study does not take the other actors participating in e-waste recycling, distributors, importers, professional users of electronic and electrical equipment, etc., into account. A second limitation of the study is its cross-sectional nature. A longitudinal study would have yielded more robust results.

## 5. Conclusions

Through this work, it is clear that stress is a serious problem among e-waste workers in the French-speaking West African region. Socioeconomic and working conditions such as insufficient income, the high number of working days per week, perceived violence at work, and the interference of work with family responsibilities and/or leisure are associated with increased stress. It then becomes imperative to address the problems of perceived stress in the e-waste sector by implementing effective public health interventions focusing on the issue of occupational stress and its corollaries. Health promotion interventions would benefit from incorporating stress reduction strategies to address health-risk behaviour among e-waste workers. We suggest that these strategies take the associated factors identified in this study into account. In addition, an occupational health management system for informal sector activities should be established by the public institutions that are in charge of labor and health in partnership with e-waste workers and/or their associations. This system should include an awareness program on stress and a program to improve the working conditions of these workers.

## Figures and Tables

**Table 1:** Sociodemographic characteristics of e-waste workers.

Variables	Countries, n (%)				
	Benin	Burkina-Faso	Ivory Coast	Mali	Senegal
Age (year), n(%)					
<25	51 (6.89)	31 (4.19)	18 (2.43)	33 (4.46)	26 (3.51)
(25–45)	86 (11.62)	102 (13.78)	87 (11.76)	78 (10.54)	71 (9.59)
(45–65)	20 (2.70)	24 (4.46)	38 (5.14)	33 (4.46)
≥65	0 (0.00)	01 (0.14)	03 (0.41)	02 (0.27)	03 (0.41)
Sex, n(%)					
Male	156 (21.08)	155 (20.95)	140 (18.92)	139 (18.78)	130 (17.57)
Female	01 (0.14)	03 (0.41)	01 (0.14)	12 (1.62)	03 (0.41)
Marital status, n(%)					
Single	63 (8.51)	59 (7.97)	51 (6.89)	50 (6.76)	51 (6.89)
Common-law marriage	75 (10.14)	26 (3.51)	73 (9.86)	0 (0.00)	0 (0.00)
Divorced	02 (0.27)	0 (0.00)	0 (0.00)	02 (0.27)	03 (0.41)
Married	17 (2.30)	73 (9.86)	17 (2.30)	99 (13.38)	79 (10.68)
Educational status, n (%)					
Primary	53 (7.16)	56 (7.57)	49 (6.62)	22 (2.97)	58 (7.84)
Lower secondary	58 (7.84)	40 (5.41)	30 (4.05)	30 (4.05)	32 (4.32)
Upper secondary	15 (2.03)	12 (1.62)	12 (1.62)	28 (3.78)	13 (1.76)
University	05 (0.68)	12 (1.62)	07 (0.95)	11 (1.49)	0 (0.00)
No formal education	26 (3.51)	38 (5.14)	43 (5.81)	60 (8.11)	30 (4.05)

Concerning the level of stress, perceived stressed showed a prevalence of 76.76% was found among e-waste workers, with 74.19% of the participants being found to have a moderate level of stress and 2.57% of them having with a high level of stress. Recyclers were the most exposed to work-related stress (79.27%), followed by repairers (76.49%), and collectors (75.49%). The proportion of workers experiencing a high level of perceived stress was more elevated in the Ivory Coast (1.5%) within workers aged 25 to 44 years old and in those working as collectors (1.32%). Detailed results are presented in Table 2.

**Table 2:** General characteristics of study participants based on level of stress.

Variables	Total	Level of Stress, n (%)		
		High	Moderate	Absent
Countries, n (%)				
Benin	157 (21.22)	07 (0.95)	124 (16.76)	26 (3.51)
Burkina Faso	158 (21.35)	02 (0.27)	116 (15.68)	40 (5.41)
Ivory coast	141 (19.05)	09 (1.22)	95 (12.85)	37 (5.00)
Mali	151 (20.41)	01 (0.14)	119 (16.08)	31 (4.19)
Senegal	133 (17.97)	0 (0)	95 (12.84)	38 (5.14)
Age (years), n (%)				
<25	159 (21.49)	02 (0.27)	123 (16.62)	34 (4.59)
(25–45)	424 (57.30)	12 (1.62)	319 (43.11)	93 (12.57)
(45–65)	148 (20.00)	05 (0.68)	99 (13.38)	44 (5.95)
≥65	09 (1.22)	0 (0)	08 (1.08)	01 (0.14)
E-waste workers category, n (%)				
Collectors	257 (34.73)	10 (1.35)	184 (24.86)	63 (8.51)
Recyclers	164 (22.16)	03 (0.41)	127 (17.16)	34 (4.59)
Repairers	319 (43.11)	06 (0.81)	238 (32.16)	75 (10.14)

**Table 3:** Association between perceived stress and selected occupational factors related to e-waste activities.

	Total	Perceived Stress		Univariate Analysis			Multivariate Analysis		
		Frequency	%	OR	CI 95%	*p*-Value	ORa	CI 95%	*p*-Value
Age (year)									
<25 years	159	125	16.89	1			-		
(25–45)	424	331	44.73	0.97	[0.62–1.50]	0.886	-		
(45–65)	148	104	14.05	0.64	[0.38–1.08]	0.094	-		
≥65 years	9	8	1.08	2.18	[0.38–41.08]	0.471	-		
Support from supervisor/workmates									
No	655	498	67.30	1			-		
Yes	85	70	9.46	1.47	[0.84–2.74]	0.196	-		
Insufficient income									
No	350	252	34.05	1			1		
Yes	390	316	42.70	1.66	[1.18–2.35]	0.004	1.46	[1.03–2.08]	0.036
Weekly working days									
Less than or equal to 6 days	498	365	49.32	1			1		
7 working days	242	203	27.43	1.90	[1.29–2.85]	0.002	1.67	[1.12–2.52]	0.014
After-work Burnout									
No	297	220	29.73	1			-		
Yes	443	348	47.03	1.28	[0.91–1.81]	0.158	-		
Exposure to adverse physical conditions									
No	302	223	30.14	1					
Yes	438	345	46.62	1.31	[0.93–1.85]	0.119	-		-
Violence in the workplace									
No	694	526	71.08	1			1		
Yes	46	42	05.68	3.35	[1.33–11.27]	0.023	3.07	[1.21–10.40]	0.036
Daily working time									
Less than 8 h	317	252	33.60	1					
More than 8 h	423	316	42.13	0.76	[0.54–1.08]	0.127	-		
Interference of work with family/leisure responsibilities									
No	582	427	56.93	1			1		
Yes	158	141	18.80	3.01	[1.81–5.31]	<0.001	2.41	[1.42–4.31]	0.002

ORa: adjusted odds ratio.

## Data Availability

The data presented in this study are available upon request from the corresponding author.
